# Risk Stratification Before and During Treatment in Newly Diagnosed Multiple Myeloma: From Clinical Trials to the Real-World Setting

**DOI:** 10.3389/fonc.2022.830922

**Published:** 2022-03-09

**Authors:** Francesca Bonello, Lorenzo Cani, Mattia D’Agostino

**Affiliations:** SSD Clinical Trial in Oncoematologia e Mieloma Multiplo, Division of Hematology, University of Torino, Azienda Ospedaliero-Universitaria Città della Salute e della Scienza di Torino, Torino, Italy

**Keywords:** multiple myeloma, newly diagnosed, clinical trials, real world, risk stratification

## Abstract

Multiple Myeloma (MM) is a hematologic malignancy characterized by a wide clinical and biological heterogeneity leading to different patient outcomes. Various prognostic tools to stratify newly diagnosed (ND)MM patients into different risk groups have been proposed. At baseline, the standard-of-care prognostic score is the Revised International Staging System (R-ISS), which stratifies patients according to widely available serum markers (i.e., albumin, β 2-microglobulin, lactate dehydrogenase) and high-risk cytogenetic abnormalities detected by fluorescence *in situ* hybridization. Though this score clearly identifies a low-risk and a high-risk population, the majority of patients are categorized as at “intermediate risk”. Although new prognostic factors identified through molecular assays (e.g., gene expression profiling, next-generation sequencing) are now available and may improve risk stratification, the majority of them need specialized centers and bioinformatic expertise that may preclude their broad application in the real-world setting. In the last years, new tools to monitor response and measurable residual disease (MRD) with very high sensitivity after the start of treatment have been developed. MRD analyses both inside and outside the bone marrow have a strong prognostic impact, and the achievement of MRD negativity may counterbalance the high-risk behavior identified at baseline. All these techniques have been developed in clinical trials. However, their efficient application in real-world clinical practice and their potential role to guide treatment-decision making are still open issues. This mini review will cover currently known prognostic factors identified before and during first-line treatment, with a particular focus on their potential applications in real-world clinical practice.

## 1 Introduction

Multiple myeloma (MM) is a clonal plasma cell malignancy characterized by strong inter-patient and intra-clonal heterogeneity, resulting in different survival rates, ranging from months up to decades (1). Several factors contribute to this heterogeneity: patient fitness, tumor burden, chromosomal and genetic abnormalities, disease localizations, and response to therapy (2, 3). It is essential to identify baseline patient-related and disease-related risk factors, in order to choose the most appropriate treatment. The Durie and Salmon Staging System was the first staging system introduced in 1975 (4); since then, several baseline staging systems have been proposed, aiming at a better risk stratification of MM patients. On the other hand, risk factors emerging during therapy are equally important and may help modulate treatment over time. Among these dynamic factors, the achievement of measurable residual disease (MRD) negativity has the strongest prognostic significance, to the point that MRD has been proposed as a surrogate for survival (5, 6). Patients failing to achieve MRD negativity show shorter remissions and survival regardless of treatment received, as compared to MRD-negative patients (7). Early relapse (ER, e.g., relapse after 12-24 months) is another strong predictor of shorter overall survival (OS), and ‘early relapsers’ remain a challenge for clinicians (8, 9).

Among the available tools to stratify the risk of MM patients, some are standardized and routinely used in clinical practice, while others are still at an experimental stage and mainly limited to clinical trials (10). Among these, genetic analyses, gene expression profiling (GEP), and circulating tumor plasma cells (CTC) are emerging as complementary to cytogenetic analyses to detect high-risk disease. New methods for MRD detection in the peripheral blood, such as cell-free DNA and mass spectrometry (MS), are gaining interest. The ultimate goal is to combine different tools to improve the risk stratification of MM patients at an experimental stage and, subsequently, in clinical practice. Nevertheless, especially for smaller centers, the standardization and implementation of some of these techniques in clinical practice could be challenging, since they are expensive and labor intensive. Therefore, the evaluation of the feasibility and applicability of each method is of utmost importance.

This mini review will describe different tools for risk assessment and stratification in MM and their potential applicability in the real-world setting.

## 2 Risk Stratification Before Starting Treatment

### 2.1 Definition of Active Multiple Myeloma

Historically, the diagnosis of active MM requiring treatment has been made in the presence of monoclonal plasma cells in the bone marrow and signs of organ damage such as hypercalcemia, renal failure, anemia, and/or bone lesions (CRAB features) (11), while the detection of bone marrow plasmacytosis ≥10% in the absence of clinical symptoms has defined a condition of asymptomatic smoldering MM (SMM). In 2014, the definition of active MM has broadened to also include asymptomatic patients with high-risk features conferring an 80% probability of evolution to symptomatic MM within 2 years and therefore requiring treatment. The updated slim-CRAB criteria included the presence of ≥60% monoclonal plasma cells in the bone marrow, a free light chain ratio ≥100 and involved free light chain ≥10 mg/dL, and the detection of at least one osteolytic lesion ≥5 mm detected by magnetic resonance imaging (MRI) (1). The distinction between SMM and active MM is not always straightforward, also considering that the clinical course of SMM is widely heterogeneous. Several risk stratification tools, which are beyond the aim of this review, have been developed over the years to help clinicians distinguish indolent “low-risk” SMM from SMM at higher risk of progression (12, 13). In clinical practice, a thorough baseline staging of patients with monoclonal gammopathies is of utmost importance to avoid treatment delays and organ damage in MM patients and overtreatment in SMM patients. Imaging techniques for the detection of early signs of MM are particularly important: in this regard, MRI is recommended in all patients with negative computed tomography (CT) or positron emission tomography/CT (PET/CT), given its higher sensitivity in detecting focal lesions (14). The evidence of increased fluorodeoxyglucose (FDG) uptake in PET/CT in the absence of osteolytic lesions is not currently considered a MM-defining event, even if it has been associated with an increased risk of progression from SMM to MM (15). In the future, genomic features and clonal patterns of evolution might help identify SMM patients with the highest risk of progression, who would therefore benefit from early treatment (16, 17). Nevertheless, it will take many years for these strategies to be validated and enter clinical practice.

### 2.2 Disease-Related Factors

The risk stratification of newly diagnosed (ND)MM patients has always relied on clinical and laboratory parameters reflecting tumor burden (4). The International Staging System (ISS) combined serum albumin and β 2-microglobulin values to stratify patients into three stages with different survival (median OS 62, 44, and 29 months for stages I, II, and III, respectively) (18). In 2015, a Revised ISS (R-ISS) was proposed and validated, adding to the ISS serum lactate dehydrogenase (LDH) and cytogenetic abnormalities (CA) detected by fluorescence *in situ* hybridization (FISH). Again, three risk groups of patients were identified with different survival (median OS not reached [NR], 83, and 43 months for stages I, II, and III, respectively, with a median follow-up of 46 months) (10). The R-ISS allowed better patient stratification and revolutionized the prognostic characterization of MM patients by combining high-risk CA – namely del(17p), t(4;14), and t(14;16) – with serum parameters. To date, the R-ISS is the gold standard for risk stratification in NDMM, and FISH analysis is routinely performed and represents a strong baseline prognostic predictor. Nevertheless, the R-ISS model can still be perfectible. Indeed, while the role of del(17p) and t(4;14) is well established, the prognostic impact of t(14;16) remains controversial (19). Recent data showed that patients harboring t(14;16) had inferior OS (median 53 months), but it is not clear whether this inferior prognosis was conferred by the translocation in itself or rather by the concomitant presence of other high-risk features, such as CA (83% patients) or ISS stage III (43%) (20). Moreover, the R-ISS classifies about 60% of NDMM patients as “intermediate-risk”, and a further stratification of this group might be useful. Besides the three CA included in the R-ISS, chromosome 1q copy-number alterations are also linked to poorer prognosis (21). Recently, a second revision of the ISS (the R2-ISS model) incorporating 1q copy-number alterations and excluding t(14;16) has been proposed. The R2-ISS model stratified patients as at “low risk”, “low-intermediate risk”, “intermediate-high risk”, and “high risk”, with different OS and PFS, thus confirming that the large group of the R-ISS intermediate-risk patients is widely heterogeneous in terms of survival (22). Recent data suggested that 1q amplification (e.g., ≥4 copies), rather than 1q gain (e.g., 3 copies) was associated with the worst outcome (23, 24). Translocations involving the immunoglobulin (Ig) L locus (particularly IgL-MYC translocations) have also been related to poor prognosis, early relapse, and resistance to immunomodulatory agents. In a recent analysis, IgL translocations were detected in approximately 10% of NDMM patients and were frequently found in hyperdiploid MM, which has traditionally been considered a favorable prognostic factor (25, 26). These data suggest that IgL translocations might identify an unrecognized high-risk subgroup, otherwise classified as at standard risk.

Patients carrying more than 1 high-risk CA (HRCA) have an even worse prognosis than patients with only 1 HRCA (21). In a large meta-analysis of 1900 patients treated with novel agents, the risk of death was significantly higher in patients with 2 HRCA (HR for OS 2.67) and 3 HRCA (HR 6.23) than in patients with a single HRCA (HR 1.55), as compared to patients without HRCA (27). Taken together, these findings could lead to an updated prognostic system allowing a more precise stratification of NDMM patients.

Aside from cytogenetics, specific gene mutations have been found to correlate with prognosis. For instance, while del(17p) is universally considered a high-risk feature, data showed an even worse outcome in patients with del(17p) and TP53 mutations (median PFS 152 months in patients without del(17p) and TP53 mutation, median PFS 53 months in del(17p) patients, and median PFS 36 months in del(17p) patients with concomitant TP53 mutation) (28). Gene expression profiling (GEP) identifies patterns of different gene expression in malignant plasma cells that correlate with prognosis. Different signatures have been studied, and two of them (SKY-92 MM profiler signature and MyPRS signature) were validated (29–31). In the Myeloma XI trial, patients with the SKY-92 high-risk signature (24%) had a significantly shorter PFS (median PFS 16 vs. 34 months, HR 2.6) and OS (median OS 37 months vs. NR, HR 3.9) than patients without a high-risk signature. The combination of high-risk CA and GEP signature identified a subset of patients with a particularly poor outcome who did not benefit from regimens based on novel agents (32). In this view, GEP and FISH/R-ISS could be combined to refine patient prognosis and guide therapeutic decisions. The ongoing PROMMIS trial (NCT02911571) is currently evaluating the impact and feasibility of this strategy. Similarly, the MUKnine trial is evaluating an intensive treatment pathway for NDMM patients who are considered at high risk due to the presence of high-risk GEP signature and CA (33). The mSMART risk stratification model combined CA, GEP, and plasma cell proliferation index to stratify patients into standard-risk, intermediate-risk, and high-risk groups (34). Nevertheless, GEP is an expensive and laborious technique, it is not available worldwide, and its implementation in clinical practice could consequently be challenging, especially for smaller centers. Moreover, the validated signatures do not overlap, and more data are needed to identify the most important set of genes related to high-risk disease.

CTC are another marker of adverse prognosis, as demonstrated by several works (35). In the FORTE trial, NDMM patients with high levels of CTC (>0.07%, assessed by flow cytometry) showed significantly inferior PFS (HR 2.49) and OS (HR 2.85), as compared with patients without high CTC (36). Nevertheless, the routine assessment of CTC has not yet been standardized and validated, mainly due to the lack of agreement about the optimal cut-off to define CTC positivity and the best technique for its detection.

Finally, the number and size of focal lesions detected at baseline by MRI have been shown to predict outcome. In a large dataset of NDMM patients, the presence of at least 3 large focal lesions >5 cm^2^ resulted in poor PFS (2.3 years) and OS (3.6 years) (37). The presence of extramedullary disease (accounting for 8-10% of patients at diagnosis) has been related to inferior outcome and should be considered as a hallmark of high-risk disease (38). Indeed, Rasche and colleagues demonstrated the presence of spatial heterogeneity in terms of genetic mutations within different lytic lesions/plasmacytomas in the same patient. In this light, different MM clones might co-exist in the same patient, some of them harboring high-risk genetic/chromosomal lesions that could be missed by biopsying only the iliac crest (39). However, performing multiple biopsies is impractical. In this light, imaging could be used to detect high-risk disease, but more data are needed, and, to date, radiological features are not included in any of the standardized risk models mentioned above.

### 2.3 Patient-Related Factors

#### 2.3.1 Transplant-Eligible Patients

High-dose therapy (HDT) followed by autologous stem-cell transplantation (ASCT) is considered the standard of care for medically fit NDMM patients (40). Transplant eligibility is determined by several factors, including chronological age, comorbidities, and organ function.

Historically, the cut-off age for ASCT adopted in trials and clinical practice was 65 years (41). Nevertheless, not all older patients are ineligible for ASCT: given the improvement in supportive care and induction strategies, in most countries the cut-off age for ASCT eligibility was raised to 70 years. Ongoing trials exploring new novel-agent combinations have been enrolling patients up to 70 years of age (e.g., EMN17/Perseus trial: daratumumab-bortezomib-lenalidomide-dexamethasone [VRd] vs. VRd; EMN24/IsKia trial: isatuximab plus carfilzomib-lenalidomide-dexamethasone [KRd] vs. KRd). Beyond 70 years of age, the feasibility of ASCT is debatable. In many centers, ASCT is potentially available for all patients with good organ function (renal, hepatic, pulmonary, and cardiac) and performance status, regardless of age (42). Recent data confirmed the feasibility of ASCT also in selected patients aged ≥75 years, with a low transplant-related mortality (1%) and a 2-year PFS and OS of 66% and 83%, respectively (43). In this subset of patients, a reduced conditioning (e.g., melphalan at 100-140 mg/m^2^) is often preferred.

Besides chronological age, organ function and comorbidities are other determinants of ASCT eligibility. The Hematopoietic Cell Transplantation Comorbidity Index (HCT-CI), which was initially developed for recipients of allogeneic stem-cell transplantation, was predictive of non-relapse mortality (6.1% vs. 3.4% vs. 1.8%, in high-risk, intermediate-risk, and low-risk patients respectively; p=0.002) and morbidity/mortality (13% vs. 9% vs. 4.7%, respectively; p<0.001) in patients undergoing ASCT as well (44). In clinical practice, although scores are not routinely performed to assess ASCT eligibility, they can be useful tools to select patients at higher risk of complications who might require careful monitoring and reduced-intensity conditioning.

#### 2.3.2 Transplant-Ineligible Patients

Transplant-ineligible, elderly patients represent a heterogeneous population in which stratification according to individual characteristics is essential to balance treatment efficacy with the risk of toxicity. Several models to stratify elderly patients according to their fitness have been proposed and validated over the years. The International Myeloma Working Group (IMWG) Frailty Score classifies elderly MM patients as fit, intermediate fit and frail according to age, comorbidities [assessed by Charlson Comorbidity Index (CCI)], and functional impairment (assessed by Katz Index of Independence in Activities of Daily Living [ADL] and Lawton Instrumental Activities of Daily Living [IADL]). Frail patients, as compared with intermediate-fit and fit patients, showed an inferior OS (3-year OS 57% vs. 76% vs. 84%, respectively; HR in frail vs. fit patients 3.57) as well as a higher incidence of non-hematologic toxicity (34% vs. 26% vs. 22%, respectively; HR in frail vs. fit patients 1.74) and of treatment discontinuation (31% vs. 21% vs. 17%, respectively; HR in frail vs. fit patients 2.21) (45).

The Revised Myeloma Comorbidity Index (R-MCI) categorized patients according to age, Performance Status (PS), frailty, lung and renal function, and cytogenetics; patients were deemed “fit”, “intermediate-fit”, and “frail” with a median OS of 10.1, 4.4, and 1.2 years, respectively (46). More recently, a simplified version of the IMWG Frailty Score (evaluating age, CCI, and PS) identified “frail” and “non-frail” patients, the former being at higher risk of death (HR for OS 1.86), toxicity (HR 1.16 for hematologic toxicities and 1.18 for non-hematologic toxicities), and treatment discontinuation (HR 1.66) (47). The UK Myeloma Research Alliance Risk Profile (MRP) combined PS, age, ISS stage, and circulating levels of C-reactive protein (CRP), thus identifying patients at low risk, medium risk, and high risk for OS (median 60 vs. 40 vs. 25 months, respectively) and early mortality (OR 2.14 for medium risk and OR 4.76 for high risk vs. low risk) (48). Biological markers of frailty, such as sarcopenia and senescence markers, are currently evaluated in clinical trials to improve the sensitivity of the available scores, but their routine use is not yet recommended due to the lack of standardization (49, 50). The IMWG frailty score currently represents the gold standard for risk stratification, and its use to guide therapeutic decisions in elderly patients is recommended in clinical practice (51). Aside from the strengths and weaknesses of the various models proposed, the common goal is to identify those patients who are able to receive full-dose regimens aimed at disease remission and those in whom avoiding toxicity and preserving quality of life should be the main treatment goals (52, 53). Although it is now widely recognized that chronological age alone is not sufficient to guide therapeutic decisions, patients aged ≥80 years (who, according to the IMWG frailty score, were determined to be frail by age only), similarly to patients who were determined to be frail due to comorbidities or functional impairment, showed a worse OS (median OS 43 vs. 77 months, HR 1.51, p=0.002) and a higher rate of treatment discontinuation (HR 2.34, p<0.001), as compared with non-frail patients (54). These data suggest that very advanced age is a hallmark of vulnerability regardless of comorbidities and functional status.

## 3 Risk Stratification During Treatment

After the start of treatment with new combinations, more than 50% of patients achieve a complete response (CR) according to the standard IMWG criteria (55). To further discriminate patients with residual disease beyond CR, new highly sensitive tools to monitor MRD inside and outside the bone marrow have been developed. Two techniques are now considered standards of care to detect MRD inside the bone marrow: next-generation flow (NGF) and next-generation sequencing (NGS) (56, 57).

NGF is a technique based on multiparameter flow cytometry that was standardized by the EuroFlow Consortium. NGF exploits the aberrant phenotype and the clonal light chains to detect residual malignant plasma cells inside the bone marrow. By using an 8-color 2-tube technique and several standardized steps for the processing and analysis of the sample, a high sensitivity (up to 10^−6^) can be achieved (58).

NGS is a molecular biology technique that exploits the unique immunoglobulin (Ig) gene rearrangement of the malignant plasma cell detected at diagnosis to track the levels of residual disease during treatment (59). The identification of a clone needs a baseline sample, and the identification rate of a trackable clone is 90-92%. In order to proceed, a bioinformatic tool and a certain degree of expertise are needed. Although many platforms using NGS to detect MRD are now available (60–63), the only platform for commercial use in MM approved by the Food and Drug Administration is clonoSEQ^®^ (Adaptive Biotechnologies, US-WA). Similarly to NGF, the maximum sensitivity with NGS-based MRD detection is 10^-6^.

Both NGF and NGS demonstrated to have a major prognostic role in MM. The clinical impact of NGF MRD negativity was validated in the pivotal work by Paiva and colleagues, in which NDMM patients achieving MRD negativity after consolidation showed a significantly reduced risk of disease progression or death, as compared to MRD-positive patients (HR for PFS 0.18; HR for OS 0.12) (64). Similar findings were observed in both the EMN02/HOVON 95 MM and FORTE trials (65, 66). The same impact on PFS was observed with NGS MRD negativity in several randomized trials (7, 67). A large meta-analysis by Munshi et al. confirmed the prognostic role of MRD (both by NGF and NGS) on PFS (HR 0.33) and OS (HR 0.45) (6). Both NGF and NGS are associated with advantages and disadvantages. With NGF, a fresh sample is needed, a baseline sample is not necessary, the turnaround time to obtain results is short (3-4 h), and the bone marrow quality can be checked in full detail. NGS can be performed on stored samples, but a baseline sample is required, the turnaround time is longer (at least 1 week), and the bone marrow quality cannot be checked. Applicability of both NGS and NGF in standard clinical practice is increasing, but remains suboptimal because these techniques are not usually available in smaller centers, and not even referral centers can always routinely assess MRD (68). Worldwide efforts are being made to evaluate the reproducibility and applicability of NGF and NGS also in the real-world setting (69).

The achievement of MRD negativity at high sensitivity predicts long-term remission/survival (70) and can potentially overcome the negative prognostic impact of high-risk features detected at diagnosis (64).

In terms of prognostic prediction, imaging techniques for the detection of residual plasma cells in focal bone lesions and extramedullary lesions outside the bone marrow are complementary to MRD techniques inside the bone marrow (71).

Compared to other imaging techniques, ^18^F-Fluorodeoxyglucose PET/CT (^18^F-FDG–PET/CT) can characterize disease lesions according to FDG uptake, making it a standard technique to identify residual plasma cells in focal MM involvements outside the bone marrow. An important step towards the standardization of PET-defined complete response was achieved by using Deauville criteria (72). More recently, diffusion-weighted (DW-)MRI has emerged as a functional technique for the detection of residual disease, and response assessment categories (RACs) have been proposed (73) and validated in a dataset of NDMM patients, in whom complete imaging response by DW-MRI after ASCT was associated with improved PFS (HR 0.28, p=0.004) (74). Although these data need validation in prospective trials before entering clinical practice, DW-MRI holds promise for MRD evaluation, since it overcomes the limitations of false negatives by PET/CT in case of non–FDG-avid lesions.

Peripheral blood can potentially contain information about residual disease from the whole body, since it comes from both the bone marrow and extramedullary sites. However, peripheral blood-based MRD techniques using NGF (75) to detect CTC and using NGS (76) to detect circulating tumor DNA after treatment failed to achieve a sensibility that was high enough to reflect the MRD status inside the bone marrow at the same time points.

Promising results were observed with novel technologies, such as mass spectrometry (MS) to measure the monoclonal protein (M-protein) secreted by the malignant plasma cells at a sensitivity that is higher than that used with serum protein electrophoresis (SPEP) and serum immunofixation (s-IFX) (77, 78). Different platforms using MS are being developed to identify M-proteins (79). MS is a non-invasive technique that can be repeated many times during the follow-up of MM patients, virtually every time the clinician needs to define response to anti-MM treatments (79).

Quantitative immunoprecipitation MS (QIP-MS) detects, at high sensitivity, the intact M-protein after a step of immunomagnetic serum enrichment using IgG/A/M, κ, λ, free κ and free λ specific beads (78). Interestingly, this technique showed a high concordance when compared to bone marrow NGF and was associated with a comparable prognostic impact in terms of PFS. Moreover, considering bone marrow NGF as a reference technique, QIP-MS showed a high negative predictive value and, as a consequence, it can be used to avoid unnecessary bone marrow aspirations (77).

## 4 Conclusion

Many new prognostic factors emerged from clinical trials, although the applicability of many of them in the real-world setting is questionable (**Table 1**). Ideally, the prognostic factors to be analyzed in all patients should (1) guide patient management, (2) be available also in non-specialized centers, (3) be cost-effective, and (4) be consistently reproducible.

**Table 1:** Risk-assessment tools and applicability in clinical practice.

Tool	Time of evaluation	Advantages in clinical practice	Limitations in clinical practice
**ISS** **- serum albumin** **- β 2-microglobulin**	Baseline	- Routinely available laboratory parameters - Easy to use - Economic - Validated	- Inadequate stratification - It does not take into account any biological data
**R-ISS** **- serum albumin** **- β 2-microglobulin** **- serum LDH** **- FISH analysis**	Baseline	- Better stratification of MM patients compared to ISS - Validated	- FISH may not be available in smaller centers - Some high-risk CA are not included in the model [e.g., amp(1q) and IgL translocations] - It does not include all high-risk phenotypes (e.g., extramedullary disease) - Most patients (60%) in the intermediate-risk group
**IMWG Frailty Score** **- age** **- Charlson Comorbidity Index (CCI)** **- ADL and IADL**	Baseline	- Gold standard to guide treatment choice in elderly patients - Additional information to chronological age only - Easily available parameters - Validated	- Time required to define CCI and ADL/IADL
**GEP**	Baseline	- Complementary information to FISH analysis - Validated signatures (MyPRS; SKY92)	- Not available in many centers - Expensive - Labor intensive - Several available GEP signatures that do not overlap
**CTC**	Baseline and during treatment	- It might be used as prognostic factor at baseline and to monitor MRD	- Lack of standardization (e.g., cut-off, technique) - Flow cytometry/NGS not available in smaller centers
**Bone marrow MRD**	During treatment	- Dynamic prognostic factor - It might guide treatment decisions - Validated techniques (NGF and NGS)	- Not available in smaller centers - Labor intensive (NGS>NGF) - Expensive - Invasive procedure - NGF requires fresh samples - NGS requires baseline samples
**^18^F-FDG–PET/CT**	Baseline and during treatment	- Dynamic prognostic factor - It might guide treatment decisions - Validated response criteria	- Expensive - Exposure to radiation - Not available in smaller centers - False negatives if non–FDG-uptaking lesions
**DW-MRI**	Baseline and during treatment	- Higher sensitivity in detecting focal lesions (baseline and residual) - Dynamic prognostic factor - No false-negative results - Validated response criteria - No radiations	- Expensive - Possible false-positive results in case of post-treatment rebound hypercellularity, G-CSF use, etc. - Acquisition and interpretation of data are labor intensive - Data about its concordance with bone marrow MRD are still limited
**Mass spectrometry**	During treatment	- Non-invasive technique for MRD assessment - Possibility to combine it with bone marrow MRD to increase sensitivity	- Not validated - Not available in many centers - Baseline samples improve data interpretation

ISS, International Staging System; R-ISS, revised ISS; LDH, lactate dehydrogenase; FISH, fluorescent in situ hybridization; MM, multiple myeloma; CA cytogenetic abnormalities; ADL, Katz Index of Independence in Activities of Daily Living; IADL, Lawton Instrumental Activities of Daily Living; GEP gene-expression profile; CTC, circulating tumor plasma cells; MRD, measurable residual disease; IMWG, International Myeloma Working Group; NGF, next-generation flow; NGS, next-generation sequencing; ^18^F-FDG–PET/CT, ^18^F-Fluorodeoxyglucose positron emission tomography/computed tomography; FDG, fluorodeoxyglucose; G-CSF, granulocyte colony-stimulating factor; DW-MRI, diffusion-weighted magnetic resonance imaging.

**Figure 1:**
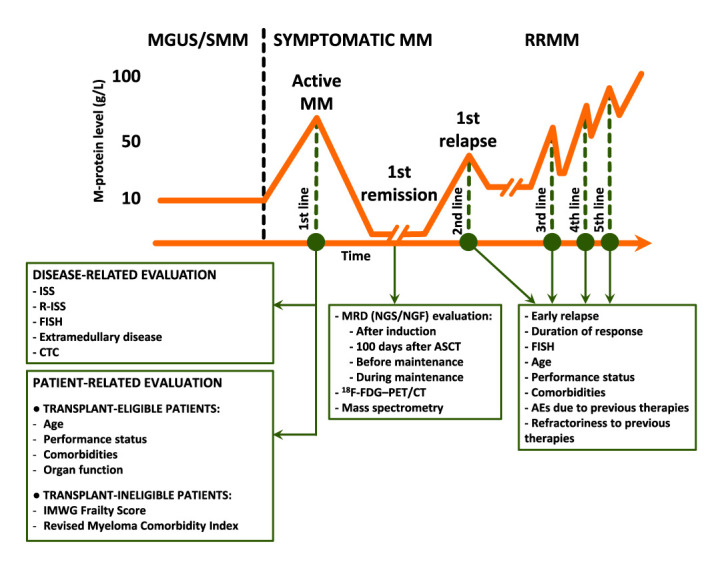
Prognostic evaluation before, during, and after therapy. M-protein, monoclonal protein; MGUS, monoclonal gammopathy of undetermined significance; MM, multiple myeloma; SMM, smoldering MM; RRMM, relapsed/refractory MM; ISS, International Staging System; R-ISS, Revised ISS; FISH, fluorescence *in situ* hybridization; CTC, circulating tumor plasma cells; IMWG, International Myeloma Working Group; MRD, measurable residual disease; NGS, next-generation sequencing; NGF, next-generation flow; ASCT, autologous stem-cell transplantation; ^18^F-FDG–PET/CT, ^18^F-Fluorodeoxyglucose positron emission tomography/computed tomography; AEs, adverse events.

The combination of baseline and dynamic tools to stratify patient risk could be the best strategy to pursue (**Figure 1**); yet the tools to be used and the timing of their use remain to be defined. At baseline, costly techniques requiring highly specialized centers (e.g., NGS, GEP, RNAseq) are less likely to spread in the real-world setting, while widely (e.g., FISH) and/or simple (e.g., flow cytometry in peripheral blood to detect CTC) techniques could be more appealing in this setting.

Patient-related factors must be considered to deliver effective treatment without inducing excessive toxicity, especially in elderly patients in whom patient-related factors are equally if not more important than disease-related factors.

During treatment, MRD evaluation is now considered the strongest predictor of patient outcome. In clinical trials, however, bone marrow evaluations are performed at multiple time points. The need to achieve a sustained MRD negativity over time and the best time points to measure it are still matters of debate. Many ongoing trials are exploring MRD-guided treatments (80), and their findings will clarify which time points are the most relevant for treatment-decision making. In the future, the use of MRD in clinical practice will follow what is important for treatment-decision making. At present, however, MRD evaluation merely plays a prognostic role in clinical practice.

In conclusion, new tools are continuously developed in clinical trials, but their applicability in clinical practice should always be simultaneously verified by acquiring more real-world data.

## Author Contributions

Substantial contributions to the conception or design: all authors. Interpretation of data: all authors. First draft: all authors. Critical revision for important intellectual content: all authors.

Final approval of the version to be published: all authors. Agreement to be accountable for all aspects of the work in ensuring that questions related to the accuracy or integrity of any part of the work are appropriately investigated and resolved: all authors.

## Conflict of Interest

Author MD has received honoraria for lectures from and served on the advisory boards for GlaxoSmithKline and Sanofi.

The remaining authors declare that the research was conducted in the absence of any commercial or financial relationships that could be construed as a potential conflict of interest.

## Publisher’s Note

All claims expressed in this article are solely those of the authors and do not necessarily represent those of their affiliated organizations, or those of the publisher, the editors and the reviewers. Any product that may be evaluated in this article, or claim that may be made by its manufacturer, is not guaranteed or endorsed by the publisher.
